# Semen parameters and fertility potency of a cloned water buffalo (*Bubalus bubalis*) bull produced from a semen-derived epithelial cell

**DOI:** 10.1371/journal.pone.0237766

**Published:** 2020-08-21

**Authors:** Monika Saini, Suman Sheoran, Kennady Vijayalakshmy, Rasika Rajendran, Dharmendra Kumar, Pradeep Kumar, Rakesh Kumar Sharma, Rajesh Kumar, Tushar K. Mohnaty, Naresh L. Selokar, Prem Singh Yadav

**Affiliations:** 1 Division of Animal Physiology and Reproduction, ICAR-Central Institute for Research on Buffaloes, Hisar, India; 2 Artificial Breeding Research Center, ICAR-National Dairy Research Institute, Karnal, India; University of Connecticut, UNITED STATES

## Abstract

Semen contains epithelial cells that can be cultured *in vitro*. For somatic cell nuclear transfer applications, it is essential to know whether clone(s) produced from semen-derived epithelial cells (SedECs) are healthy and reproductively competent. In this study, the semen and fertility profile of a cloned bull (C1) that was produced from a SedEC were compared with its donor (D1) and with two cloned bulls (C2, C3) that were produced from commonly used skin-derived fibroblast cells (SkdFCs). We observed variations in some fresh semen parameters (ejaculated volume and mass motility), frozen-thawed sperm parameters (plasma membrane integrity, and computer-assisted semen analysis (CASA) indices), but values are within the normal expected range. There was no difference in sperm concentration of ejaculated semen and frozen-thawed semen parameters which include sperm motility, percentage of live and normal morphology sperm, and distance traveled through oestrus mucus. Following *in vitro* fertilization (IVF) experiments, zygotes from C1 had higher (*P < 0*.*05*) cleavage rates (81%) than C2, C3, and D1 (71%, 67%, and 75%, respectively); however, blastocyst development per cleaved embryo and quality of produced blastocysts did not differ. The conception rate of C1 was 46% (7/15) and C2 was 50% (8/15) following artificial insemination with frozen-thawed semen. Established pregnancies resulted in births of 7 and 6 progenies sired by C1 and C2, respectively, and all calves show no signs of phenotypical abnormalities. These results showed that semen from a cloned bull derived from SedECs is equivalent to semen from its donor bull and bulls cloned from SkdFCs.

## Introduction

Somatic cell nuclear transfer (SCNT) methods provide an opportunity to multiply superior bulls which could be used to disseminate desirable genotypes and phenotypes for accelerating the speed of genetic improvement strategies [1]. Previously, cattle and buffalo bulls have successfully been cloned using SCNT methods [2–6]. To produce cloned bulls, fibroblast cells cultured from skin-tissue biopsies have commonly been used as nuclear donors; however, epithelial cells available in semen can also be used [7–9]. Also, semen is a non-invasive donor cell source that could safeguard precious elite bulls from septic infection, which may result from tissue biopsy injuries [9]. According to our information, there is only one study that reported the successful birth of cloned bulls using semen-derived epithelial cells (SedECs) [5]. Information is lacking whether clone(s) produced from SedECs are healthy and reproductively as competent as non-cloned bulls and bulls that were cloned from commonly used skin-derived fibroblast cells (SkdFCs). Therefore, information on the reproductive status of such clone(s) is required to explore possibilities to use SedECs to produce multiple clones of bull(s).

The contribution of a bull to genetic improvement depends on the production of quality frozen semen with good fertility. Typically, a bull’s fertility is estimated by a large number of artificial inseminations in females, which is an expensive and time-consuming task [10]. Semen parameters, including the computer-assisted sperm analysis (CASA), the ability to produce embryos through *in-vitro* fertilization (IVF), and conception rate following limited artificial insemination (AI), have been examined to assess the potential fertility of cloned bulls [3, 4, 11, 12]. A recent study in water buffalo (*Bubalus bubalis*) reported that ejaculate volume, sperm concentration, CASA indices, and conception rate following AI did not differ between a cloned bull and its donor [6]. Similar observations have also been reported in cattle [3, 4, 11, 12]. Cloned bulls, including buffalo, examined in these previous studies were produced from SkdFCs. Here we investigated the semen parameters, development and quality of IVF embryos, and offspring production ability of a cloned water buffalo (*Bubalus bubalis*) bull that was produced from a semen-derived epithelial cell.

## Materials and methods

### Animal derivation and ethics

In the present study, three cloned Murrah buffalo bulls (C1, C2, and C3), of which C1 was produced from a semen-derived epithelial cell [5], whereas, C2 [6] and C3 [13] were produced from SkdFCs, were used to examine semen and reproductive characteristics. All cloned bulls used in this study were produced through the handmade cloning method [14]. D1 represents a donor of C1, D2 represents a donor of C2; whereas, we do not have a donor of C3 since it was produced from a fibroblast cell of a slaughterhouse fetus. The semen of C1 and C3 was collected and cryopreserved at the ICAR-National Dairy Research Institute (NDRI), Karnal, India; whereas, semen of C2 was collected and cryopreserved at the ICAR-Central Institute for Research on Buffaloes (CIRB), Hisar, India. Cloned and donor bulls were housed and managed under similar farm conditions at their respective centers. Frozen semen doses used in this study were produced when C1 was 28 months old, C2 was 24 months old, C3 was 29 months old, D1 was 32 months old, and D2 was 29 months old. This study was approved by the Institute Animal Ethics Committee (IAEC-CIRB/19-20/A/006) and efforts were made to minimize pain and suffering during the handling of bulls and female buffaloes and a minimum number of female buffaloes were used to complete this study. All procedures were performed according to the ethical standards of the institutions.

### Semen production and cryopreservation

We collected semen from cloned and donor bulls using the artificial vagina method and processed according to guidelines of the minimum standard protocol for bovine semen cryopreservation in India (http://www.dahd.nic.in/guidelines/large-ruminantsbovine). From each bull, semen was collected twice a week. After collection, initial semen parameters such as volume, mass motility, and sperm concentration were recorded for qualifying semen for cryopreservation. The ejaculated semen was collected in a graduated test tube that gives the semen volume. The percentage of mass motility was recorded using a phase-contrast microscope at 400X magnification and sperm concentration calculated using an Accucell bovine photometer (IMV, L’Aigla, France). Semen has a volume of greater or equal to 2 ml, sperm concentration above 500 million/ml, and mass motility above 65 percentages were processed for cryopreservation. The qualified semen samples were cryopreserved according to a method described previously [15, 16]. In brief, ejaculated semen was diluted in egg yolk based extender containing tris (3.02%, w/v), citric acid (1.67%, w/v), fructose (1%, w/v), egg yolk (20%, v/v), penicillin (1000 IU/ml), streptomycin (1000 μg/ml) and glycerol (6.4%, v/v) to make a final concentration of 80 million sperm/ml. The diluted semen was kept for 3–4 h for equilibration at 4°C in a cold cabinet. Thereafter, semen was loaded into 0.25 ml plastic straws (20 million sperm/straw), cooled down from 4 to −140°C using a programmable biological freezer (Mini Digi-cool, IMV Technologies, L’Aigle, France) and subsequently cooled straws were plunged into liquid nitrogen for storage. Frozen semen straws from three different freezing days were transported in a cryocan from the semen production center of NDRI and CIRB to our laboratory for further analysis.

### Assessment of frozen-thawed semen parameters

In each experiment, three random straws from each freezing day were thawed in a water bath at 37°C for 45 seconds. Immediately after thawing, the percentage of post-thaw sperm motility was recorded using a phase-contrast microscope at 400X magnification. The frozen-thawed semen parameters such as Eosin-Nigrosin staining-based sperm viability (%) and normal morphology (%), hypo-osmotic swelling test-based membrane integrity (%) and cervical mucus penetration ability were estimated by a single operator as described previously [17, 18]. For analysis, at least 150 sperm were examined per slide and five slides were examined per experiment and three independent experiments were performed.

#### Sperm viability and morphology

Twenty μl of frozen-thawed semen was placed in a corner of a pre-warmed glass-slide (37°C) and smear was prepared. The smeared slides were dipped in Eosin–Nigrosin solution (1.7 gm of Eosin, 10 gm of Nigrosin was dissolved in 100 ml distilled water) for 5 min. Following incubation, the slides were rinsed to remove excess stain and allowed to dry. Stained slides were evaluated under an oil immersion phase-contrast microscope at 1000X magnification. Sperm that remained unstained were classified as live and sperm that stained pink or red were classified as dead. The morphological defects such as double-head sperm, elongated head, detached head, proximal droplet, distal droplet, bent tail or midpiece, and coiled tail or midpiece were also recorded. The percentages of viable and normal morphology sperm were calculated separately.

#### Plasma membrane integrity

Plasma membrane integrity was evaluated using the hypo-osmotic swelling test as described previously [15]. In brief, 10 μl of frozen-thawed semen was incubated in 1 ml of hypo-osmotic solution (0.73 gm sodium citrate 2H2O and 1.35 gm fructose in 100 ml distilled water) for 60 min at 37°C. Following incubation, 20 μl of the sample was placed under a coverslip on a warm slide (37°C), and sperm’s tail coiling was assessed under a phase-contrast microscope at 400X magnification. The sperm that had coiled tails were considered having intact plasma membranes, and percentage data were calculated.

#### Cervical mucus penetration ability

Cervical mucus was aseptically collected from buffaloes in estrus and was aspirated into 9-cm long graduated glass capillaries. One end of a capillary was sealed with petroleum jelly and another end was left open. The open end of capillary was dipped in 0.1 ml frozen-thawed semen in a 1.5 ml centrifuge tube and incubated for 45 mins at 37°C. Following incubation, distance traveled (in mm) by sperm was measured under a phase-contrast microscope at 100X magnification. Five capillaries were examined to calculate migration distance.

#### CASA indices

The sperm motility indices were analyzed using a CASA system (IVOS12.1, Hamilton-Thorne Biosciences, Beverly, MA, USA) as described previously [15]. Before the analysis of CASA-based sperm indices, the frozen-thawed semen was diluted in tris buffer that gives 40X10^6^ sperm/ml. One μl of diluted semen was loaded in a Leja slide (8 chambers with 20μm depth) and 5 optical fields were analyzed by software. The motility and kinetic parameters such as total motility (TM, %), straight linear velocity (VSL, μm/s), average path velocity (VAP, m/s), curvilinear velocity (VCL, μm/s), average lateral head displacement (ALH, μm/s), beat cross frequency (BCF, Hz), straightness (STR,%) and linearity (LIN,%) were recorded. The CASA software settings, including frame rate, frames acquired, VAP cut-off, and STR cut-off, were identical for all experiments.

### IVF embryo production and quality of produced embryos

For assessment of fertilization ability of cloned bull semen, IVF experiments (n = 3) were performed as described previously [19]. In brief, ovaries were collected from an abattoir, and oocytes were obtained by aspiration of antral follicles using 18-gauge needles. The oocytes having over three layers of cumulus cells and homogenous ooplasm were washed three times with the washing medium (TCM-199 supplemented with 10% fetal bovine serum (FBS), 0.81 mM sodium pyruvate, and 50 μg/ml gentamycin) and twice with the *in-vitro* maturation medium (TCM-199 supplemented with 10% FBS, 5 μg/ml porcine FSH, 1 μg/ml estradiol-17β, 0.81 mM sodium pyruvate, and 50 μg/ml gentamycin). For *in-vitro* maturation (IVM), oocytes (15–20 oocytes per droplet) were placed in 100 μl droplets, which covered with sterile mineral oil, in a 35 mm culture dish (4 droplets per dish) and cultured for 24 hrs in a 5% CO_2_ incubator at 38.5°C. Following IVM, matured oocytes were subjected to IVF. The two semen straws were thawed as described in the above section. Thawed semen was washed twice through centrifugation at 1000g for 5 mins in the Bracket and Oliphant's medium which was supplemented with 10 mg/ml fatty acid-free bovine serum albumin (BSA), 10 μg/ml heparin, 137.0 μg/ml sodium pyruvate, and 1.9 mg/ml caffeine sodium benzoate (BO medium). Then, the sperm pellet was re-suspended in 0.5 ml of the BO medium (4x10^6^ sperm/ml). The matured oocytes were rinsed once with BO medium and transferred to 50 μl droplets (15–20 oocytes/droplet) of BO medium in a 35 mm culture dish. The 50μl of sperm suspended in BO medium was added to 50 μl droplets having oocytes and incubated for 20 hrs in a 5% CO_2_ incubator at 38.5°C. After incubation, gentle washing was given to oocytes to remove expanded cumulus cells. The denuded oocytes were washed three times with TCM-199 medium supplemented with 10% FBS to remove dead sperm. Presumptive zygotes were then cultured in 100 μl droplets of Research Vitro Cleave medium (K-RVCL-50; Cook) supplemented with 1% fatty acid-free BSA in a 35 mm culture dish (15–20 embryos per droplet) for 7 days in a 5% CO_2_ incubator at 38.5°C. After 48 hrs of culture, presumptive zygotes were evaluated under an inverted microscope at 100X magnification for cleavage, which was considered as an indicator of successful fertilization. To access the quality of produced blastocysts, the terminal deoxyribonucleotidyl transferase-mediated dUTP–digoxigenin nick end-labeling (TUNEL) staining was performed as described previously [20]. Six to eight IVF blastocysts of each bull were used to determine the total cell number and the apoptotic index.

### Artificial insemination

Frozen-thawed semen of C1 and C2 was used to inseminate 15 and 16 cyclic female buffaloes, respectively. Buffaloes that completed three-parity were used for AI. One insemination was performed in buffaloes that showed the natural sign of estrus. Because of the inadequate availability of recipient female buffaloes, we could not perform AI using clone C3, and donors (D1 and D2). Pregnancies were determined by ultrasonography on day 30 of AI and reconfirm again on day 60.

### Statistical analysis

The data, except conception rates, were tested for normality and homogeneity of variances using SPSS 20 software (IBM SPSS Statistics for Windows, Version 20.0, IBM Corp Armonk, NY) before running subsequent analysis. The Student’s test was used to compare between cloned bulls and their respective donors (C1 vs D1, and C2 vs D2). The comparisons between clones (C1 vs C2 vs C3) were analyzed by one-way ANOVA with the Fisher LSD post hoc test, except conception rates. Differences were considered significant if the p-value was < 0.05.

## Results

### Fresh and frozen-thawed semen parameters

Clone C1 and C2 had higher ejaculated semen volume than C3. C1 had higher semen volume than D1, whereas there was no difference in the semen volume of clone C2 and D2. Between clones, C1 had the lowest mass motility and membrane integrity, which were also lower than D1 (Table 1). There were no differences in sperm concentration of ejaculated semen and post-thaw parameters, such as sperm motility, and percentage of live or normal morphology sperm. Eosin-Nigrosin staining results showed that 75 to 80% of sperm were viable and more than 80% of sperm have normal morphology. Cervical mucus penetration assay showed that sperm from cloned and donor bulls traveled 25 to 30 mm distance through oestrus mucus.

**Table 1:** Semen parameters of cloned buffalo bulls and donors.

Murrah buffalo bull		Pre-freezing parameters*			Post-freezing parameters^$^				
		Ejaculate volume (mL)	Sperm concentration (10^6^ /mL)	Mass motility, %	Post-thaw sperm motility (%)	Live sperm (%)	Cervical mucus penetration distance (mm)	Morphological normal sperm (%)	Membrane integrity (%)
Clones	C1	4.5±0.5^a^	853.3±53.3	66.6±1.6^b^	61.6 ± 1.7	83.9±4.2	28.0±1.7	87.0±2.4	40.7±7.0^b^
	C2	5.0±0.5^a^	988.7±143.8	75.0±2.0^a^	61.6 ± 1.6	79.0±6.1	28.0±4.3	83.4±2.6	57.0±3.2^a^
	C3	3.0±0.0^b^	933.3±70.5	75.0±2.8^a^	56.6 ± 4.4	80.5±4.1	25.0±0.5	84.8±3.6	55.6±4.4^a^
Donors	D1	3.6±0.6^b^	933.3±133.3	73.3±1.3^a^	60.0 ± 2.8	77.5±5.1	29.6±3.2	84.2±2.1	55.5±2.4^a^
	D2	5.1±0.6^a^	1002.1±113.5	76.2±2.4^a^	56.6 ± 1.6	81.7±5.2	27.3±2.9	87.7±2.2	45.7±6.1^b^

Values with the same column without common superscript letters (a-b) differ (P<0.05).

*Data generated from five semen collections

^$^ Data generated from three replicates, in each replicate, three straws from each freezing day were used.

### CASA indices

The CASA-based motility and kinetic parameters are shown in Table 2. The VSL, VAP, BCF, STR, and LIN of C1 were significantly higher; whereas, the VCL and ALH were lower than that of C2 and C3. Between clones, TM is highest in C3. Clone C1 has significantly lower VAP, VSL, VCL, ALH, and TM; whereas, the BCF, STR, and LIN were higher than that of D1. Clone C2 has significantly lower VAP and BCF than that of D2. There were no differences in other CASA indices.

**Table 2:** CASA indices in frozen-thawed semen of cloned bulls and donors.

Murrah buffalo bull		CASA variables of frozen-thawed semen							
		VAP (μm/s)	VSL (μm/s)	VCL (μm/s)	ALH (μm)	BCF (Hz)	STR (%)	LIN (%)	TM (%)
Clone	C1	97.6 ± 0.3^a^	86.2 ± 0.5^b^	146.1 ± 1.5^b^	5.1 ± 0.1^b^	38.7 ± 0.3^b^	85.7 ± 0.2^c^	60.6 ± 0.5^b^	35.7 ± 0.7^a^
	C2	96.0 ± 1.1^a^	80.0 ± 1.9^a^	173.7 ± 1.7^a^	7.4 ± 0.1^a^	29.3 ± 0.6^a^	79.6 ± 0.5^a^	44.8 ± 0.5^a^	32.5 ± 1.0^a^
	C3	92.6 ± 2.1^b^	77.3 ± 1.1^a^	153.5 ± 1.8^c^	5.8 ± 0.1^c^	35.8 ± 0.3^c^	81.8 ± 0.4^b^	55.4 ± 0.6^c^	51.7 ± 1.1^b^
Donor	D1	112.7 ± 0.9^c^	93.6 ± 0.8^c^	187.3 ± 1.5^d^	7.3 ± 0.1^a^	32.8 ± 0.2^d^	81.8 ± 0.2^b^	51.0 ± 0.4^d^	40.8 ± 1.1^c^
	D2	99.1 ± 2.1^a^	80.6 ± 2.6^a^	176.2 ± 4.2^a^	7.2 ± 0.1^a^	32.9± 0.4^d^	80.7 ± 0.8^ab^	46.7 ± 0.6^a^	37.2 ± 0.6^a^

Data generated from three replicates, in each replicate, five frozen semen straws from each freezing day were used. Values with the same column without common superscript letters (a-c) differ (P<0.05).

### *In vitro* fertilization rate and embryo quality

Frozen-thawed semen of cloned and donor bulls was used to produce IVF embryos, and resultant presumptive zygotes were allowed to develop to blastocysts. We used the cleavage rate as an indicator of the *in vitro* fertilization ability of sperm. Between clones, C1 had a higher cleavage rate (81%) than C2 and C3 (71% and 67%, respectively). The cleaved embryos that subsequently developed to blastocysts were not significantly different (Table 3). The TUNEL staining results showed that the total cell number and the apoptotic index of blastocysts were not different (Table 3).

**Table 3:** *In Vitro* Fertilization (IVF) and Artificial Insemination (AI) using frozen-thawed semen of cloned bulls and donors.

Murrah buffalo bull		*In vitro* embryonic development			Quality of IVF blastocyst		AI success		
		Oocytes, n	Cleaved embryos, n (%)	Developed Blastocysts, n (%)	Total cell number	Apoptotic index	Inseminated buffaloes, n	Pregnant buffaloes, n (conception rate, %)	Live calves born
Clone	C1	174	141 (81.0)^a^	14 (10.0)	148.8 ± 19.4	10.0 ± 1.0	15	7 (46)	7
	C2	160	115 (71.8)^b^	12 (10.4)	179.3 ± 10.6	9.6 ± 1.9	16	8 (50)	6
	C3	250	168 (67.2)^c^	15 (8.9)	163.2 ± 14.5	11.0 ± 0.6	-	-	-
Donor	D1	208	156 (75.0)^b^	15 (9.6)	154.5 ± 19.9	10.2 ± 2.0	-	-	-
	D2	189	130 (68.7)^c^	12 (9.2)	172.0 ± 18.6	12.0 ± 3.5	-	-	-

Data generated from five IVF replicates. In each IVF replicate, frozen-thawed semen of one cloned bull and his donor was used. IVF trails of C3 were conducted independently. The blastocyst rates were calculated from cleaved embryos. Values with the same column without common superscript letters (a-c) differ (P<0.05).

### Conception rate following AI

Because of the limited availability of recipient buffaloes, we could perform a few AIs using frozen-thawed semen of C1 and C2. The conception rate of C1 was 46% (7/15) and C2 was 50% (8/15) using single insemination. These established pregnancies resulted in births of 7 and 6 progenies sired by C1 and C2, respectively.

## Discussion

Previous studies in farm animals reported that SedECs can be used as nuclear donors to produce cloned embryos [7–9]; however, there is only one study that reported the successful births of cloned bulls using SedECs [5]. SedECs, when cultured from semen (fresh, stored, or even cryopreserved), can provide an opportunity to breeders to restore superior bulls into breeding schemes through SCNT methods [9]. In this regard, the semen parameters and fertility potency of bull(s) that have been cloned using SedECs need to be thoroughly investigated. This study is a first report that examined the semen parameters and fertility potency of the cloned bull produced from SedEC. Although some variations were observed, the overall semen parameters such as ejaculated volume, sperm concentration, mass motility, post-thaw parameters (motility, percentage of live and normal morphology sperm, mucus penetration ability, membrane integrity, and CASA indices), IVF and AI data showed that the SedEC cloned bull (C1) was as reproductively competent as its donor (D1) and bulls (C2 and C3) that were cloned from commonly used SkdFCs.

The bull C1 was produced through the handmade cloning method at NDRI, Karnal in 2013 [5], and now 7 years old and healthy. The C1 bull had been raised on similar farm conditions that provided to other non-cloned bulls, including its donor D1. For semen production, the standard bull screening procedures were followed, and C1 was trained to produce semen. We also included two cloned bulls (C2 and C3) that were produced using SkdFCs. The bulls, C1, C2, and C3, started producing semen at 20, 19, 22 months, respectively, which is within an expected age (20 to 30 months) to start semen production in Murrah buffalo (unpublished data of Artificial Breeding Research Center, NDRI, Karnal). Similar observations have been reported that the average age to start semen collection was similar between cloned goats and their donors [21].

We observed variations in some fresh semen parameters such as ejaculated volume, sperm concentration, and mass motility (Table 1), but the values were within the expected range of values reported previously in Murrah buffalo bulls [22]. Similar variations in ejaculated semen volume, sperm concentration, and percentage of motile sperm have been reported in cattle bulls which were produced using embryo splitting [23], blastomere separation [24], and SCNT methods [4, 11]. In agreement with the earlier report [11], the ejaculated semen volume of clone C1 was higher than that of its donor D1. However, the sperm concentration of C1 was similar to D1 and other clones (C2 and C3), this is an agreement with earlier reports that sperm concentration of cloned cattle bulls, bucks, boars, cat and buffalo bulls were similar to their donors [4, 6, 21, 25, 26]. Despite C1 having the lowest mass motility compared to its donor (D1) and other cloned bulls (C2 and C3), but it was within the expected range [22].

Ejaculated semen of clone C1 was successfully cryopreserved, which shows that C1 sperm withstand the ultra-low temperature of the cryopreservation process. The frozen-thawed semen parameters such as sperm motility, cervical mucus penetration ability, and percentages of live and normal morphology were similar between clones and their respective donors. However, the C1 has the lowest membrane integrity among all examined bulls. The sensitivity of C1 sperm to the cryopreservation process may cause the lowest membrane integrity. Similar, individual differences in sperm parameters have been reported in previous studies in cattle [12], pig [25], and cats [26]. The computerized technique, CASA, has been used in farm animals to study sperm motility and kinetic parameters to predict the potential fertility of bulls [10]. We observed that there were differences in examining CASA parameters (TM, VCL, ALH, BCF, STR, and LIN) between clones and donors. Differences in CASA indices have also been reported between cloned cattle bulls and their donors [11, 12, 27]. These differences can be because of the age of bull at the time of semen collection, individual response to the cryopreservation process, the season of semen freezing, environmental factors, and laboratory-to-laboratory variations [11].

Over the years, several *in-vitro* methods have been developed to predict bull fertility. Besides sperm motility and functional parameters, the *in-vitro* fertilization ability of sperm can also be used for predicting bull fertility [28]. AI is an expensive and time-consuming task and also requires sufficient females. IVF does not suffer from these drawbacks; therefore, many bulls can be tested for a different aspect of their semen quality [28]. We observed that fertilization ability (based on cleavage rates) of C1 thawed sperm appeared to be higher than that of its donor D1 and other cloned bulls (C2 and C3); whereas, blastocyst development from cleaved embryos and quality of produced blastocysts did not differ. The higher CASA indices, namely BCF, STR, and LIN in the sperm of C1 could have contributed to the higher cleavage rate; however, this effect could not translate to the higher blastocyst development rate and better blastocyst quality (total cell number and apoptotic index of blastocyst). Similar results were also reported that the frozen-thawed sperm of a cloned bull had higher cleavage rates, but the blastocyst production rate and quality of blastocysts were not different [11]; they assessed the quality of blastocysts through a ratio of inner cell mass cells and trophectodermal cells. Recently, a study in water buffalo also reported that there was no difference in terms of blastocyst production rate following IVF with frozen-thawed sperm of a cloned Murrah bull and its donor [6], although they did not examine cleavage rates and quality of produced blastocysts. This study had low blastocyst rates (~10%), which is in agreement with other IVF studies in buffalos [29–32]. The laboratory-to-laboratory variations, conditions, protocols, and other factors could have contributed variation in IVF results [33].

Although the IVF can provide a hint about the bull’s fertility; however, the complex nature of sperm and variability in IVF protocols prevents the absolute use of the IVF method as a choice for predicting the potential fertility of bulls [28]. AI and natural mating are the only accurate methods that have been used to prove that bulls can produce offspring [28]. Due to the limited availability of female buffaloes, we could perform AI using frozen-thawed semen of two cloned bulls, namely C1 and C2. We found that the conception rate following AI with C1 frozen-thawed semen was comparable to C2 semen, 46%, and 50% respectively. Conception rates achieved in this study were within the normal expected range (30–60%) in water buffalo [34]. Recently, it had also reported that there was no difference in terms of conception rates following AI with frozen-thawed sperm of a cloned Murrah bull and its donor [6]. The pregnancies of C1 and C2 have resulted in the births of 7 and 6 progenies (1–2 months old), respectively, and these progenies had normal gestation periods, birth weights, and growth rate (data not shown). Results presented in this study and by other groups suggested that once cloned bulls matured well and produce freezable semen, the clones could produce normal progenies through AI, IVF, and embryo transfer [4, 6, 11, 27]. This study opened a new avenue to produce clones of superior bulls using SedECs. However, this study results need to be interpreted carefully, since we presented only one bull data, and conducted a few experiments.

In conclusion, the present study demonstrated that the cloned bull (C1) produced from the semen-derived epithelial cell produced freezable quality semen. Variations observed in frozen-thawed semen parameters including CASA indices did not affect the blastocyst development rate following AI and conception rate following AI. Production of progenies using frozen-thawed semen showed that C1 can be used as a sire to produce offspring. Further, more studies need to be conducted to confirm the suitability SedECs in SCNT applications.

## Supporting information

S1 Data (XLS)
